# Prevalence and route of transmission of undiagnosed human immunodeficiency virus infection among children using provider-initiated testing and counselling strategy in Ido-Ekiti, Nigeria: a cross-sectional study

**DOI:** 10.11604/pamj.2019.34.62.9374

**Published:** 2019-10-01

**Authors:** Oluwaseyi Tosin Babatunde, Layi Solomon Babatunde, Susan Modupe Oladeji

**Affiliations:** 1 Department of Paediatrics, Benjamin Carson Senior School of Medicine, Babcock University, Ilishan-Remo, Ogun State, Nigeria; 2 Department of Community Medicine, Benjamin Carson Senior School of Medicine, Babcock University, Ilishan-Remo, Ogun State, Nigeria; 3 Department of Ear, Nose and Throat, Benjamin Carson Senior School of Medicine, Babcock University, Ilishan-Remo, Ogun Sate, Nigeria

**Keywords:** PITC strategy, prevalence, paediatric HIV

## Abstract

**Introduction:**

Ninety-one percent of global Human Immunodeficiency Virus (HIV) infection in children occurs in sub-Saharan Africa. Provider Initiated Testing and Counselling (PITC) Strategy is a means of reducing missed opportunities for HIV exposed or infected children. The present study determined the prevalence of HIV infection using PITC Strategy among children seen at the Paediatric Emergency Unit of Federal Medical Centre (FMC), Ido-Ekiti, and the possible route of transmission.

**Methods:**

Cross-sectional study on prevalence of HIV infection using PITC model. 530 new patients whose HIV serostatus were unknown and aged 15 years or below were recruited consecutively and offered HIV testing. Serial algorithm testing for HIV infection using Determine HIV-1/2 and Uni-Gold rapid test kits was adopted. Seropositive patients younger than eighteen months had HIV Deoxyribonucleic Acid Polymerase Chain Reaction (HIV DNA PCR) test for confirmation.

**Results:**

Twenty-four (4.5%) of the 530 patients were confirmed to have HIV infection; of whom 19 (79.2%) were less than 18 months of old; with age range of 5 to 156 months. Fifteen (62.5%) of the infected children were females; likewise, the gender specific infection rate was higher (%) among the females compared with (%) among the males. Two of the HIV infected children’s mothers were late, while the remaining 22 mothers (%) were HIV seropositive. Mother-to-child-transmission was the most likely route of transmission in the children.

**Conclusion:**

PITC strategy is vital to the early diagnosis and effective control of HIV infection in children. However, this cannot be totally effective if PMTCT is not optimized.

## Introduction

HIV counselling and testing (HCT) is a key intervention for HIV prevention and a critical entry point into life-sustaining treatment and care programme for those infected with the virus [1]. Knowledge of HIV status is critical to expanding access to timely HIV treatment, care and support that offers PLWHA an opportunity to receive information and tools to prevent HIV transmission to others. Furthermore, HCT has been noted to have the potential to encourage openness, hence contributing to the reduction of fear and stigma in society [2]. Health facilities represent a key point of contact with people infected with HIV. Evidence from both industrialized and resource-constrained settings suggests that many opportunities to diagnose and counsel individuals at health facilities exist [3,4]. In Australia, a review of records at a Canberra Sexual Health Centre showed that more than half of HIV infected patients with delayed diagnosis had earlier been in health facilities especially the emergency unit, and almost all of these individuals had at least one risk factor that should have prompted healthcare providers to test and counsel for HIV infection [3]. A study in Uganda showed that about half of adult who were offered HIV testing at a hospital were subsequently found to be HIV infected and 83% were unaware of their HIV status, even though 88% had been to a health unit in the previous six months [4]. Concerned by persistent late diagnoses of HIV infection and a high proportion of people with HIV who are unaware of their HIV status, WHO and UNAIDS in 2007 issued a guidance on PITC for health facilities. The PITC strategy refers to HIV testing and counselling which is initiated by healthcare providers to persons attending healthcare facilities as a standard component of medical care provided to the patient, regardless of whether the patient shows sign and symptoms of HIV infection or not [5]. Walensky *et al.* [6] in a prospective study conducted in three hospital-associated urgent care centres and one emergency department in Massachusetts got a prevalence of 2.0% of undiagnosed HIV infection using PITC as against 1.9% using self-referral testing. A prospective study by Basset *et al.* [7] in an out-patient department in South Africa showed that routine HIV testing leads to significantly higher rates of detection of HIV infection. The policy statement on HIV Testing published by UNAIDS and WHO recommends that HIV testing and counselling be offered to all children seen in paediatric health services in generalized epidemic settings especially in acute care settings like emergency units [5]. It is hoped that children with HIV infection will be picked early thereby facilitating linkage to treatment programme. Studies using the PITC Strategy in Paediatric Units in Nigeria are few [8]. Most studies have been conducted in antenatal care settings. The present study therefore set out to use the PITC strategy to determine the prevalence of undiagnosed HIV infection among children seen at Paediatric Emergency Unit (PEU) of Federal Medical Centre (FMC), Ido-Ekiti, and the possible route of transmission.

## Methods

**Study design:** the study was a prospective, hospital-based, descriptive cross-sectional study.

**Study setting:** it was carried out at the Paediatric Emergency Unit of FMC, Ido-Ekiti over a period of six months (April-September 2012); a tertiary hospital that serves as a referral centre for the neighbouring towns in Ekiti State and neighbouring Ondo, Osun and Kwara States. It is a 180-bedded hospital that runs general and specialist clinical services in twenty departments. There is an ongoing HIV infection treatment, care and support programme in the hospital. The Paediatric Emergency Unit is a 13-bedded ward with about 100 patients seen monthly (unpublished data).

**Study participants:** the subjects were consecutive new paediatric patients with unknown HIV serostatus, aged 0-15 years who presented in the PEU with any illness. The patients were recruited after signing or thumb-printing an informed consent form by the parents/caregivers. The assent of the patients who were seven years and older was sought by explaining the purpose of the study and details of the sample collection to them in a manner they would understand. Patients with documented HIV status at presentation were excluded from the study. Each patient was recruited once until the desired sample size was attained.

**Sample size determination:** the minimum sample size required for the study was determined using the formula:

$$n=Z^{2}P\left(1-P\right)/d^{2}$$_[9]._

P = 0.5 (no similar study had been done in the region); d = 0.05. The estimated minimum sample size was 385. However, a total of five hundred and thirty patients were however tested.

**Data collection:** caregivers and patients were given HIV pre-test counselling using WHO guideline on PITC with the choice of “opting out” [5]. Counselling cards for paediatric HIV disclosure adopted by Paediatric AIDS Treatment for Africa were used to obtain assent from children aged seven years and above. Pre-test and post-test information was provided in the individual sessions. An interviewer-administered questionnaire designed for the study was used to record information from each caregiver. The socio-economic classification as described by Oyedeji [10] was adopted for the present study to determine the socio-economic status of each patient.

**The testing protocol for HIV infection:** rapid test kits used for the HIV test were determine and Unigold. The HIV test was carried out and read by the researcher at the side-laboratory attached to the PEU according to the manufacturer’s instructions. Serial algorithm testing for HIV infection was used [11]. The test results were thereafter communicated to each of the parents and/or caregivers by the researcher during post-test counselling. Patients that were aged below 18 months who were reactive to the rapid tests had Dried Blood Spots (DBS) taken immediately for HIV DNA PCR for confirmation of HIV infection. Parents/caregivers of patients aged below 18 months that were reactive to the rapid tests were advised to await confirmation by HIV DNA PCR before the final result was communicated to them. They were however managed as HIV-exposed infants until their HIV status was determined. This took a maximum period of one month. Only those that had positive HIV DNA PCR results were taken to have HIV infection.

**Patient management:** patients that had HIV infection were immediately enrolled into the paediatric HIV/AIDS treatment, care and support programme at the hospital for full evaluation and treatment as recommended by the National Guidelines for the treatment of Paediatric HIV infection in Nigeria [12].

**Data analysis:** the data were entered into a personal microcomputer and analysed using the software, Statistical Package for Social Sciences (SPSS) version 15.0 Inc. Chicago, Illinois-USA. Categorical variables were expressed in proportions, ratios and percentages, while statistical test was done using Chi-square (χ^2^) test. Statistical significance was set at p-value less than 0.05.

**Ethical consideration:** Institutional Ethical Approval was obtained from the Ethics and Research Committee of FMC, Ido-Ekiti. A written informed consent form detailing the study purpose, benefit, and possible risks to participants and their caregivers was duly signed by each caregiver. In addition, assent was obtained from children aged seven years and above who were in stable clinical condition.

## Results

A total of 530 patients consisting of 296 (55.8%) males and 234 (44.2%) females participated in the study. The ages of the patients ranged between one day and 180 months, with a median age of 14 months. More than half (59.8%) of their caregivers were in social class III (Table 1). Twenty-seven (5.1%) of the 530 patients were HIV seropositive using the rapid tests, five (18.5%) of whom were aged 18 months and above, and therefore considered to have HIV infection. The remaining 22 patients had HIV DNA PCR test for confirmation. Nineteen (86.4%) of the 22 seropositive patients below the age of 18 months were confirmed to have HIV infection, while three (13.6%) were HIV-exposed but not confirmed to have HIV infection by their HIV DNA PCR test results. All the patients who were older than nine months were confirmed to have HIV infection. Thus 24 (4.5%) out of the 530 patients were confirmed to have HIV infection, giving an overall prevalence of undiagnosed HIV infection of 4.5%. Among the children that tested positive for HIV, more than half (62.5%) were females, likewise more than half (58.3%) were less than 12 months old. Majority of them had caregivers in social class III while none had caregivers in social class V (Table 2). Table 3 shows variations in the prevalence of undiagnosed HIV infection among the study participants with socio-demographic characteristics. The prevalence was twice as high (6.4%), among the females compared with (3.0%) among the males Likewise, it was highest among those < 24 months of age being 6.6% and 7.2% in those < 12 months and those aged 12 - < 24 months respectively. Children whose parents were in social class III had the highest prevalence while it was least among those in social class V. None of these variations was statistically significant. There was a statistically significant association between HIV status and the need for admission among the children. Most (95.8) of the HIV infected children required hospital admission compared with 64.8% among the non-infected children; p = 0.004 (Table 4). The possible routes of HIV infection/exposure to HIV infection were explored. These included blood transfusion, history of intramuscular (IM) injections, sexual exposure and circumcision. However, none of these was found to be significantly associated with the HIV status of the study participants (Table 5). Outcome of the HIV testing of mothers of HIV infected children showed that all the 22 (91.7%) mothers who were tested had HIV infection. The other two (8.3%) mothers were dead and thus could not be tested. Twenty-one (95.5%) of the mothers with HIV infection were unaware of their HIV status and none was exposed to Prevention-of-Mother-To-Child-Transmission (PMTCT) programme. None of the mothers of the children who were not HIV infected had HIV testing done. All the 24 patients with HIV infection had had contact with healthcare workers at the immunization clinics and 10 (41.7%) of them had actually completed their immunization according to National Programme of Immunization prior to the illness that brought them to the hospital.

**Table 1:** Socio-demographic characteristics of study participants

Variable	Frequency N = 530	Percentage
**Gender**		
Female	234	44.2
Male	296	55.8
**Age (in months)**		
0 – < 12	212	40.0
12 – < 24	83	15.7
24 – < 36	48	9.1
36 – < 48	57	10.8
48 – < 60	33	6.2
>60	97	18.3
**Social class**		
I	54	10.2
II	113	21.3
III	312	58.9
IV	32	6.0
V	13	2.5
Unknown	6	1.1

**Table 2:** Distribution of HIV infected children by gender, age and parental social class

Variable	Frequency n = 24	Percentage
**Gender**		
Female	15	62,5
Male	9	37.5
**Age (in months)**		
0 – < 12	14	58.3
12 – < 24	6	25.0
24 – < 36	-	-
36 – < 48	2	8.3
48 – < 60	1	4.2
>60	1	4.2
**Parental social class**		
I	1	4.1
II	4	16.7
III	18	75.0
IV	1	4.1
V	-	-

**Table 3:** Prevalence of undiagnosed HIV infection by socio-demographic characteristics

Variable	HIV infected n = 24	HIV non-infected n = 506	Total n = 530	Prevalence (%)	95% CI	P value
**Gender**						
Female	15 (62.5)	219 (43.2)	234 (44.2)	6.4	3.92 - 10.31	0.064
Male	9 (37.5)	287 (56.7)	296 (55.8)	3.0	1.61 - 5.68	
**Age (in months)**						
0 – < 12						0.198
12 – < 24	14 (58.3)	198 (39.1)	212 (40.0)	6.6	3.97 - 10.77	
24 – < 36	6 (25.0)	77 (15.2)	83 (15.7)	7.2	1.64 - 12.76	
36 – < 48	-	48 (9.5)	48 (9.1)	-	-	
48 – < 60	2 (8.3)	55 (10.9)	57 (10.8)	3.5	1.27 - 8.27	
> 60	1 (4.2)	32 (6.3)	33 (6.2)	3.0	2.82 - 8.82	
Social class	1 (4.2)	96 (19.0)	97 (18.3)	1.0	0.98 - 2.98	
I						0.986
II	1 (4.2)	53 (10.5)	54 (10.2)	1.9	1.74 - 5.54	
III	4 (16.7)	109 (21.5)	113 (21.3)	3.5	0.11 - 6.89	
IV	18 (75.0)	294 (58.1)	312 (58.9)	5.8	3.21 - 8.39	
V	1 (4.2)	31 (6.1)	32 (6.0)	3.1	2.91 - 9.11	
Unknown	-	13 (2.6)	13 (2.5)	-	-	
	-	6 (1.2)	6 (1.1)	-	-	

**Table 4:** Admission status of HIV infected and non-infected children

Admission status	Number (%) with HIV infection n = 24	Number (%) without HIV infection n = 506	Total (%) n = 530	HIV prevalence (%)	95% CI
Admitted	23 (95.8)	328 (64.8)	351 (66.2)	6.6	4.4 - 9.64
Not admitted	1 (4.2)	178 (35.2)	179 (33.8)	0.6	0.1 - 3.1

χ^2^ = 8.514; df = 1; p = 0.004 with Yates’s correction

**Table 5:** Exploration of possible route of HIV infection in relation to HIV infection status of patient

Possible route of HIV infection	Number (%) with HIV infection N = 24	Number (%) without HIV infection N = 506	*p* Value
**Blood transfusion**			
Yes	1 (4.2)	36 (7.1)	*X* ^2^=0.664;df=2;p=0.717
No	23 (95.8)	463 (91.5)	
Unknown	0 (0.0)	7 (1.4)	
**History of IM injections**			
Yes	2 (8.3)	37 (7.3)	*X* ^2^=0.590;df=2;p=0.745
No	21 (87.5)	459 (90.7)	
Unknown	1 (4.2)	10 (2.0)	
**Sexual exposure**			
Yes	0 (0.0)	0 (0.0)	Not applicable
No	24 (100.0)	506 (100.0)	
Circumcision			
Yes	9 (37.5)	215 (42.5)	*X*^2^=0.236;df=1;p=0.785
No	15 (62.5)	291 (57.5)	

## Discussion

The prevalence of HIV infection has been shown to vary with locality and population subgroups. The prevalence of undiagnosed HIV infection among new patients in the present study using PITC Strategy was 4.5%. This was close to the overall national prevalence of 4.1% which also employed PITC Strategy though in a different clinical setting [13]. It was however higher than the prevalence rate of 1.4% documented for Ekiti State [13] where the present study was conducted. This may be due to the fact that the HIV prevalence rate in the present study was obtained in a hospital-based study conducted in patients who were ill compared with the otherwise healthy pregnant women attending antenatal clinics in Ekiti State [13]. Earlier reports on prevalence rate of HIV infection from Africa had shown higher figures than the finding from present study [14, 15]. Most of the studies had employed the use of clinically directed criteria for screening for HIV infection which might have contributed to the high prevalence rates reported. In 1996, Lucas *et al.* [15] reported a seroprevalence rate of 20% among Ivorian patients obtained from a necropsy report. That was a much higher figure compared with the 4.5% obtained in the present study. The relatively high mortality rate among patients with HIV infection might have accounted for the high prevalence of HIV infection observed in their study; since it was conducted among cadavers of paediatric patients. On the other hand, Bakaki *et al.* [14] in 2001 reported a prevalence rate of 19.2% from Uganda among patients with clinical suspicion of immune-suppression. This may partly be a reflection of the fact that patients were selected on the basis of clinical suspicion of HIV infection in their study (bias population); and the fact that Uganda has a high national prevalence of HIV infection [16].

The prevalence rates of paediatric HIV infection in earlier reports from Nigeria were also higher than the prevalence rate in the present study. Akpede *et al.* [17] in 1997 reported a prevalence of 8.6% among patients on hospital admission in the North-eastern Nigeria. Likewise, Emodi *et al.* [18] in 1998 reported a prevalence of 20% from Enugu, Ojukwu and Ogbu [19] reported prevalence of 13.7% from Abakaliki while Adejuyigbe *et al.* [20] reported a prevalence of 23% from Ile-Ife in 2003. These were all hospital based studies using serological tests. The higher prevalence rates in these earlier studies may be due to the fact that the studies were conducted among high risk groups already suspected to have immune-suppression. The use of serological tests only in these studies could have accounted for some false positive results in patients aged below 18 months. In the present study, confirmatory testing was done for patients aged below 18 months using HIV DNA PCR thereby excluding false positive cases using only Determine and Unigold rapid tests. Though the number was small, false positive results were documented only in patients under age of nine months suggesting that serological tests were probably adequate in patients older than nine months. This needs to be confirmed in a larger number of patients. Oniyangi *et al.* [21] observed a prevalence of 5.7% among paediatric patients admitted into the National hospital in Abuja. This was slightly lower than the prevalence amongst the patients who needed to be admitted in the present study. Difference in methodology may partly be responsible for the difference in the finding. The observed difference may also be due to the fact that screening was done based on clinical criteria as established by WHO [22] in their study while all patients were screened for HIV infection using laboratory test in addition in the present study. The finding in the series by Angyo *et al.* [23] however contrast sharply with the finding in the present study. They found a very low prevalence rate of 1.5% among all patients on hospital admission. This may be due to the fact that their study was retrospective; and only patients diagnosed with AIDS were screened thus some asymptomatic patients may have been missed. The PITC Strategy was employed recently to study the prevalence of HIV infection in paediatric clinical settings [16, 24, 25]. Using PITC Strategy, Kankasa *et al.* [25] found a prevalence of 29.2% among patients on hospital admission in Zambia, Wanyenze *et al.* [16] found a prevalence of 15% among similar patients in Uganda while Rogerson *et al.* [25] reported a prevalence of 18.9% among Malawian patients on hospital admission. These were all prospective studies. These were much higher than the prevalence rate of 6.6% amongst patients who were admitted into the hospital in the present study. The observed higher prevalence rates may be a reflection of the higher prevalence of National HIV infection in these countries compared to Nigeria. Previous reports from Nigeria by Ogunbosi *et al.* [8] in Ibadan, Ejiofor *et al.* [26] in Awka and Olatunji *et al.* [27] in Lagos, gave prevalence rates of 10%, 5.8% and 4.52% respectively. The HIV prevalence rate by Ogunbosi *et al.* [8] was higher than the finding from the present study. This may be due to the fact that all patients that presented in the hospital, including those referred with known HIV status during their study period were included. Moreover, Oyo State is known to have a higher HIV seroprevalence rate compared to Ekiti State. Though the study by Olatunji *et al.* [27] from Lagos was restricted to paediatric patients with haemoglobinopathy, their finding was only slightly lower than findings in the present study. Ejiofor *et al.* [26] from Awka also screened all patients presenting in their health facility during the period of their study.

The differences between the prevalence rates in the previous reports and findings in the present study may have also been influenced by the documented HIV prevalence in pregnant women attending antenatal care settings in the various locations since studies have shown that a higher proportion of paediatric HIV infection is acquired through MTCT. The prevalence of HIV infection using PITC Strategy as compared with clinical criteria based-screening tends to be lower because relatively low risk populations are being screened. The role of gender as a risk factor for MTCT of HIV is not clear [18]. The male to female sex ratio was 1:1.7 among patients with HIV infection in the present study as compared with 1.3:1 for the overall study population. Although the difference was not statistically significant. It is consistent with the findings from studies by Adejuyigbe *et al.*, [20] Oniyangi *et al.* [21] and Ogunbosi *et al.* [8] which all reported slight female preponderance. This might suggest that the female might be at higher risk of acquiring the infection, even at this early age. However, studies from other parts of Africa have however reported a slight male preponderance [18, 28]. The study population in Adejuyigbe, Oniyangi, and Ogunbosi studies were 3 days to 17 years, 6 weeks to 9 years, 0 to 15 years respectively. Children with HIV infection develop the disease manifestations early in life with more rapid course than adults. In the present study, over 95% of the patients with HIV/AIDS were five years and below with 20 (83.3%) being aged 24 months and below. Consistent with the age distribution of the HIV infected children found in our study, are the findings in the study by Spira *et al.* [29] among patients with HIV infection in Rwanda and Emodi *et al.* [18] also who reported that all patients with vertical transmission were found to be symptomatic by the age of two years in keeping with the rapid disease progression in patients without early diagnosis and initiation of HAART [18]. Although MTCT of HIV infection has been identified as the most important route of exposure of children to HIV infection, there seems to be variations in rates by demographics in MTCT route of HIV infection in Nigeria. The probable route of transmission in 22 (91.7%) of the patients with HIV infection in the present study was by MTCT. This can be deduced from the proportion of the mothers of the HIV infected children who were HIV positive and the non-involvement of any of them in measures to prevent MTCT. All the 22 mothers of the infected children had HIV infection. It should also be noted that the remaining 8.3% (two mothers) who were not screened were dead, and reported to have died of chronic illnesses. Although the mothers of children who were not HIV infected in this study were not offered HIV testing to provide statistical evidence, the possibility of mother to child transmission as the source of HIV infection among the infected children is very high. This is also supported by the age distribution of the HIV infected children; majority (83.3%) of whom were less than two years old. Though other risk factors for HIV infection such as blood transfusion and intramuscular injection were present in some of the infected and non-infected children, they were not significantly associated with the HIV status of the children. However, gene mapping is needed to ascertain whether it is the same virus seen in the children that is the same in their mothers because they were not followed from birth. Earlier report by Emodi *et al.* [18] in 1998 indicated that a lower proportion (30%) of HIV infection was due to MTCT. This may have been due to a relatively higher blood transfusion rate of 68% found in their series and the fact that their study was undertaken when routine screening for HIV in donor blood was not widely available. Some other reports from different parts of Nigeria however have attributed higher proportions of the infection to MTCT. Oniyangi *et al.* [21] Ugochukwu *et al.* [28] Adejuyigbe *et al.* [20] and Angyo *et al.* [23] reported 93.02%, 79.7%, 78.6% and 69.6% respectively. The slight variations may reflect the relative contribution of other modes of transmission in the different parts of the country where these studies were conducted.

These findings reflect the importance of maternal HIV infection in paediatric HIV infection in Nigeria and therefore paediatric HIV control measures. It is therefore instructive that twenty-one (95.5%) of the 22 mothers of the patients with HIV infection were newly diagnosed after the primary diagnosis of HIV infection in their children in the present study. The diagnosis of HIV infection in a child usually leads to the diagnosis in a parent with unsuspected HIV infection as it was the case in the present study. Such a discovery would enhance the linking of mothers to HIV infection prevention and treatment programme. While this is complementary in the effort at combating HIV/AIDS in Nigeria, a situation where most infected mothers are detected before or during pregnancy is most desired. The contribution of blood transfusion to the prevalence of HIV infection has been documented [20, 23, 28]. Blood transfusion associated HIV infection is less common than MTCT; often occurring in settings without routine screening of blood products for HIV; especially in the resource poor settings. One (4.2%) of the patients with HIV infection had a history of previous blood transfusion at a government hospital three months prior to the detection of his HIV infection status. Both parents however had HIV infection. A gene mapping is however needed to ascertain if it is by MTCT or through blood transfusion. This brings to fore the need for routine pre-transfusion screening of recipients for HIV before blood transfusion. This is in contrast with findings by Emodi *et al.* [18] Ugochukwu *et al.* [28] Adejuyigbe *et al.* [20] and Angyo *et al.* [23] that reported rates of 68%, 16.4%, 14.3% and 8.9% respectively. The sexual route of transmission of HIV is not common in paediatric patients. This was confirmed in the present study in which none of the patients with HIV infection acquired it through the sexual route. Sexual mode of transmission has been documented mostly among sexually active adolescents. Angyo *et al.* [23] and Adejuyigbe *et al.* [20] reported sexual transmission in 17.4% and 4.8% respectively in their studies. In the present study, one adolescent boy had HIV infection, most probably through the MTCT route. This was because his mother had HIV infection and there was no other identifiable route for HIV transmission and he was not sexually active. This was a likely case of slow progression. Missed opportunities for determining the HIV infection status of persons with HIV infection have been recognized as a barrier to initiate early treatment and care. All the patients with HIV infection in the present study had had at least an earlier contact with a healthcare worker at the immunization clinic; while about 40% of them had actually completed their immunization according to National Programme of Immunization prior to the discovery of their HIV infection status in the present study. Routine application of PITC in clinical setting would probably have provided an earlier diagnosis for those with severe infection before the advanced stage. Paediatric HIV infection has become a common risk factor for hospital admission [8]. Twenty-three (95.8%) of the patients with HIV infection required admission at presentation as compared with 328 (64.8%) of the patients without HIV infection. This is in consonance with reports from other parts of Africa which documented that patients with HIV infection had significantly higher admission rates varying from 1.5% to 29% of patients admitted into the hospital [8]. Selection of the ill patients for HIV screening test on admission into the hospital will be a cost-benefit criterion for screening the sick patients for HIV infection. There may be a need to make it a must approach to be used for screening patients for HIV infection. However, implementation of PITC strategy in all health service units remains the optimal approach in the prevention and control of HIV infection.

## Conclusion

The prevalence of HIV infection among patients presenting to the Paediatric Emergency Unit of the hospital was high with the predominant route of transmission being MTCT. It is recommended that Provider-Initiated Testing and Counselling Strategy be offered to all children presenting in health facilities; especially immunization clinics, under 5 well child clinics and also other wards that admits children. This will provide opportunity for early diagnosis and treatment in order to reduce the high mortality in children with HIV infection.

### What is known about this topic

- Provider initiated testing and counselling for HIV leads to significantly higher rates of detection of new cases of HIV infection compared with screening based on clinical suspicion;
- Maternal to child transmission is the most common route of HIV infection in children;
- In Nigeria, Ekiti state has one of the least prevalence rates based on the sentinel survey among pregnant women attending ANC clinics in 2010.

### What this study adds

- The prevalence of undiagnosed HIV infection among children presenting in an emergency care setting in Ekiti state, South-western Nigeria is higher than the prevalence rate documented among pregnant women attending ANC clinics in the same study area;
- All HIV seropositive infants above nine months were confirmed HIV infected using HIV DNA PCR test;
- Majority of the HIV infected children were less than two years.

## Competing interests

The authors declare no competing interests.
